# Toward a Refined Definition of Monocyte Subsets

**DOI:** 10.3389/fimmu.2013.00023

**Published:** 2013-02-04

**Authors:** Loems Ziegler-Heitbrock, Thomas P. J. Hofer

**Affiliations:** ^1^Comprehensive Pneumology Center – EvA Study Center, Helmholtz Zentrum Muenchen – German Research Center for Environmental HealthGauting, Germany

**Keywords:** classical monocytes, non-classical monocytes, intermediate monocytes, monocyte subsets, monocyte classification

## Abstract

In a nomenclature proposal published in 2010 monocytes were subdivided into classical and non-classical cells and in addition an intermediate monocyte subset was proposed. Over the last couple of years many studies have analyzed these intermediate cells, their characteristics have been described, and their expansion has been documented in many clinical settings. While these cells appear to be in transition from classical to non-classical monocytes and hence may not form a distinct cell population in a strict sense, their separate analysis and enumeration is warranted in health and disease.

## Identification of Intermediate Monocytes

**Figure 1:**
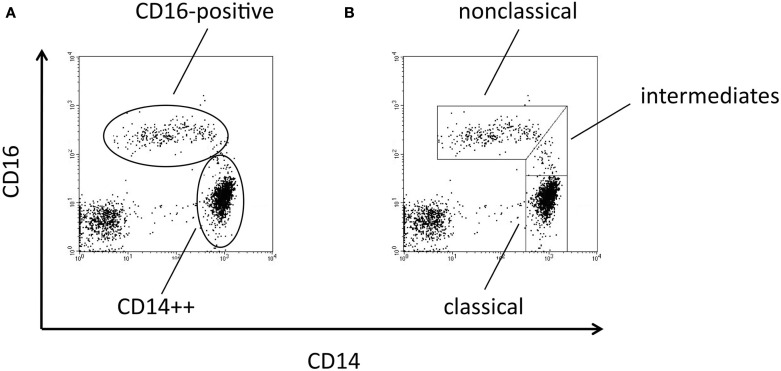
**Dot blot histograms from monocyte subpopulations using CD14 and CD16 markers**. In **(A)** the conventional gating strategy is shown, representing CD14++ and CD16-positive populations. In **(B)** a refined gating approach is given, which defines the intermediate monocytes in addition to classical and non-classical monocytes.

Human monocyte subsets are typically analyzed in multi-color flow cytometry using antibodies to CD14 (the LPS receptor) and CD16 (the low affinity receptor for IgG; Passlick et al., 1989). A dot blot histogram with the two markers demonstrates monocytes with high CD14 expression and a population of cells with co-expression of CD16 and of CD14 at a low level. The major population of CD14++ monocytes accounts for about 90% while the CD16-positive cells account for about 10% of all monocytes (Figure 1A)^1^. In the past, the very few cells with high CD14 and low CD16, which are located between the two subsets (intermediates in Figure 1B), have been allotted variously to the CD16-positive or the CD14++ monocytes (compare Figure 1A and Figure 1B). However, over the years these intermediate monocytes (Figure 1B) have gained recognition and they have been defined as a separate cell population.

**Figure 2:**
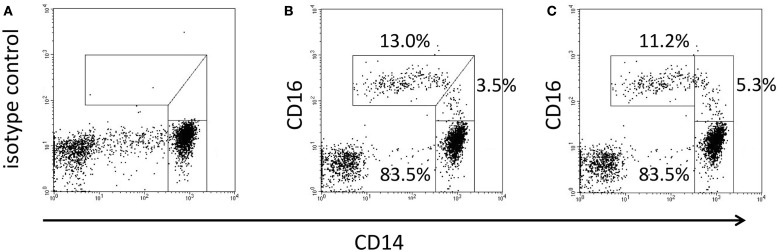
**Dissection of intermediates from classical and non-classical monocytes**. In **(A)** the isotype control defines the separating line between intermediates and classical monocytes. In **(B)** the dissection of intermediate and non-classical monocytes is done with an oblique line to the right of the cloud of non-classical events. In **(C)** a straight vertical line is placed to the left hand end of the classical monocytes.

For definition of the intermediate monocytes these cells need to be dissected from the non-classical and the classical monocytes. The latter is straightforward since an isotype control can be used to define the gating line (see Figures 2A,B).

What is more difficult is to draw the line between intermediates and non-classical monocytes. Some investigators use an oblique line such that the entire cloud of events of the CD14+CD16++ is enclosed (see Figure 2B), while others just draw a vertical line to the left of the CD14 staining of the classical monocytes (Figure 2C). While this latter approach has the advantage that it can be executed automatically it cuts into the non-classical monocytes. For an optimum separation of intermediate monocytes versus non-classical monocytes additional markers should be evaluated for defining the proper gate. Intermediate monocytes to date have been analyzed in man and in non-human primates. There is evidence that they also exist in other species like the mouse and here their definition will likely also benefit from additional unique markers.

## Cellular and Molecular Studies

Recently Wong et al. (2011) divided the intermediate monocytes further into three sub-regions of increasing CD16 expression and demonstrated a continuum in staining for a whole set of markers including CD64, CD115, CD163, CCR2, and CX3CR. Also, microarray studies showed intermediate transcript levels for almost 90% of all highly expressed genes. There was, however, some evidence for unique properties in that *CLEC10A*, *GDNF* family receptor alpha 2, and MHC class II genes were specifically high in the intermediates compared to the classical and non-classical monocytes (Wong et al., 2011).

Zawada et al. (2011) used SuperSAGE analysis and focused on genes, which were selectively increased in the intermediates. This included MHC class II genes and a whole series of additional genes like *FAU*, *SECTM1*, *CTSB*, and *RHOB*. Also they noted that the mRNA for *TIE2* (TEK, angiopoietin-2 receptor) was not significantly increased in the intermediates but antibody staining showed a specific twofold higher expression for TIE2 protein.

These data show that delineation of monocyte subsets cannot be restricted to the transcript level but will have to take on board all the other levels of gene expression including the proteome.

The Zawada findings of high TIE2 protein expression in the intermediates is in line with earlier work by Murdoch et al. (2007) and recent studies by Shantsila et al. (2011) and this indicates that cells previously termed *TIE2*-expressing monocytes strongly overlap with and may be identical to the intermediate cells.

There have been transcriptome studies into intermediate monocytes in non-human primates by Kim et al. (2010) and they reported that among the 18 genes specifically up-regulated, the matrix metalloproteinase-1 mRNA was found >50-fold higher in the intermediates versus both the classical and the non-classical monocytes. The three studies looking at the transcriptome of intermediate monocytes in man and in non-human primates found different genes to be selectively expressed in these cells. This may be due to different technologies or to heterogeneity in gene expression among the different (out-bred) individuals. Still, the data on selective gene expression suggest that the intermediate monocytes may play distinct functional roles in immune response and inflammation.

## Functional Studies

Most functional studies into monocyte subsets have looked at two types of cells (classical and non-classical) with intermediate monocytes being excluded or allotted to one or the other of the two main monocyte types. However, a few studies specifically addressing functions of the intermediates have been published.

Looking at cytokine production Skrzeczynska-Moncznik et al. (2008) isolated intermediate monocytes based on CD14 and CD16 expression and they demonstrated highest IL-10 production in these cells in response to LPS and to zymosan.

Earlier functional studies by Grage-Griebenow et al. (2001) defined the intermediate monocytes as CD64+ CD16+ cells and demonstrated in this subset the highest capacity to present antigen to T-cells. Here the intermediates were superior with respect to antigen-specific induction of IL-12 and IFNgamma and with respect to induction of alloantigen-induced T-cell proliferation. Zawada et al. (2011) confirmed this in showing that the intermediates have a slightly higher capacity to induce superantigen-mediated T-cell proliferation.

Also, studies in an angiogenesis *in vitro* model revealed a selective cluster formation for the intermediate monocytes. Together with the selective increase of TIE2 protein expression and a similar increase of endoglin and VEGF receptor 2 by the intermediates (Zawada et al., 2011) this suggests a specific role in angiogenesis for these cells. In line with this concept, work on *TIE2*-expressing monocytes in the mouse has shown that among blood mononuclear cells only these cells are capable of inducing a capillary network (De Palma et al., 2005).

Monocytes are precursors for dendritic cells and macrophages. For the non-classical monocytes a higher propensity to become DCs with a higher capacity to induce T-cell proliferation and IL-4 in CD4 T-cells has been reported (Randolph et al., 2002; Bajana et al., 2007). Also recently it was demonstrated that macrophages generated from CD16-positive monocytes (isolated by no-touch procedures) maintain a differential expression pattern and show higher phagocytosis activity when compared with macrophages, which were derived from the classical monocytes (Frankenberger et al., 2012). It is still unclear whether the intermediate monocytes just represent a transitional type of cell or whether they are a clearly distinct cell population. One way to address this is to purify the CD14++CD16+ intermediate monocytes and determine whether with *in vitro* culture they develop into non-classical monocytes or into a unique progeny, which is different from the progeny of classical or non-classical monocytes. If it can be demonstrated that intermediate monocytes give rise to a unique type of DC or macrophage then this would support the concept that they are a biologically meaningful monocyte subpopulation.

## Clinical Studies

In clinical settings the intermediate monocytes have been noted to increase in number in several diseases (for a detailed review, see Wong et al., 2012). In asthma a pronounced increase of the intermediates was seen in moderate and severe forms (Moniuszko et al., 2009). When such patients were challenged with inhalation of allergen, the numbers of intermediates decreased, and the patients responded with bronchoconstriction (Kowal et al., 2012). This would be in line with a scenario, in which upon allergen challenge the intermediates migrate into the lung and contribute to bronchoconstriction.

For rheumatoid arthritis Cooper et al. (2012) noted a strong increase of intermediates with a concomitant decrease of classical monocytes. They also showed that high levels of the intermediates predicted reduced response to therapy. In patients with colorectal cancer the percentage of intermediate monocytes was found increased and this was more pronounced in local than in metastatic disease (Schauer et al., 2012).

In adult survivors of childhood acute lymphoblastic leukemia intermediate monocytes were found increased along with increases of several inflammatory markers (Sulicka et al., 2013) and this was suggested to contribute to the increased atherogenesis seen in these patients. In fact, intermediate monocytes were found to predict cardiovascular events in dialysis patients (Heine et al., 2008), which have a high risk of such events (reviewed in Heine et al., 2012). The predictive power of these cells was confirmed by the same team in a general at-risk population (Rogacev et al., 2012). Also, intermediates were found to be elevated in ST-elevation myocard infarct with a peak on day 1 post the event (Bajana et al., 2007; Tapp et al., 2012). Also, in stroke patients the intermediates were reported to increase by day 2 and they were predictive of subsequent infections (Urra et al., 2009). Furthermore, the same study demonstrated that the numbers of these monocytes were inversely related to mortality, i.e., high numbers of intermediate monocytes went along with better survival. Such acute and dramatic events like a myocard infarct or a stroke are associated with a stress response and studies into non-classical monocytes have shown that catecholamines can lead to an expansion of these cells (Steppich et al., 2000; Kittner et al., 2002). It remains to be shown whether catecholamines play a role in the increase of intermediate monocytes in stroke or MI, as well.

When testing M-CSF in preclinical trials Weiner et al. (1994) have noted a strong expansion of non-classical monocytes and it was evident in those early studies that initially there was an expansion of the intermediate monocytes. Also, histograms shown by Saleh et al. (1995) suggest an expansion of the intermediates by M-CSF. Conversely, anti-M-CSF treatment was recently shown to deplete both non-classical and intermediate monocytes in man (Korkosz et al., 2012). When these cells recover over a 4-week period of time then first the intermediate monocytes reappear followed by the non-classical monocytes (Korkosz et al., 2012).

**Figure 3:**
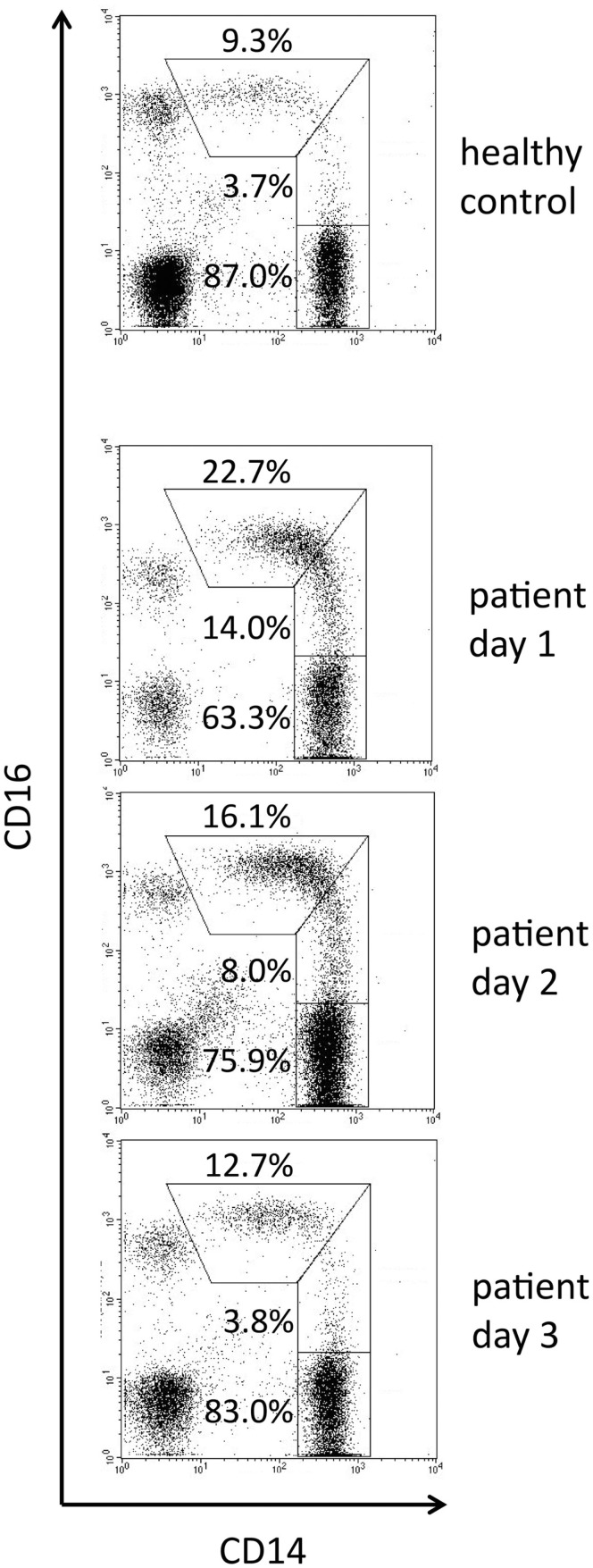
**Time course of monocyte subpopulations during natural infection**. Blood samples were taken from a control donor and on consecutive days from a patient with skin infection by beta-hemolytic streptococci group A (erysipelas). First symptoms of lower limb inflammation occurred on day 2, day 1 is the day of admission to hospital. The flow cytometry setting was such that a high signal to noise ratio for CD16 allowed for clear display of the intermediate monocytes. Taken from thesis work of Alexia Horelt, Faculty of Medicine, Ludwig-Maximilians University of Munich, 2003. Other parts of the thesis have been published in Horelt et al. (2002).

In a model of viral infection, injection of the TLR7/8 ligand R-848 into non-human primates was shown to lead to a strong increase in intermediate monocytes on day 1 followed by a peak of non-classical monocytes on day 2 (Kwissa et al., 2012). These data also support the transitional nature of the intermediate monocytes. Another example illustrating this transition in the setting of a natural bacterial infection in a patient is seen in Figure 3. Here the day 1 sample shows an increase of the intermediate cells compared to control with most CD16-positive events located in the area of the line which in healthy individuals separate intermediates and non-classical monocytes. On day 2 cells shift further to the left and the window for intermediate monocytes clears up. Finally, on day 3 intermediates are back to normal and the CD16-positive monocytes have their peak in the center of the non-classical window.

Whether or not the intermediate monocytes, induced by various stimuli, go on to develop into non-classical monocytes may be dependent on the type and on the strength of the stimulus.

When comparing gene expression patterns in classical and non-classical monocytes for man versus mouse it was noted that a differential gene expression between the two subsets was reverse in man and mouse for a number of biologically relevant genes (Ingersoll et al., 2010). Still, the mouse can serve as a model for human blood monocyte subsets. While the intermediate monocytes have been described in the mouse (Sunderkötter et al., 2004), the potential of this model for the analysis of these monocytes still needs to be exploited.

## Concluding Remarks

Since the discovery of the CD16-positive monocytes by flow cytometry this approach has been used to distinguish these cells from the major population of classical monocytes. A third population of intermediate monocytes initially had been overlooked because of its low number and transitional nature. Now clinical studies have demonstrated that these cells can strongly expand and laboratory analyses have shown intermediate but also unique features for these cells. Specific markers for the intermediate monocytes will be required in order to unequivocally identify and enumerate these cells.

## Conflict of Interest Statement

The authors declare that the research was conducted in the absence of any commercial or financial relationships that could be construed as a potential conflict of interest.

## Footnotes

^1^+ Represents a fluorescence signal that is clearly separated from the negative control staining, ++ is a signal about 10-fold (1 log) stronger than +.
